# Rapid and Differential Diagnosis of Sepsis Stages Using an Advanced 3D Plasmonic Bimetallic Alloy Nanoarchitecture‐Based SERS Biosensor Combined with Machine Learning for Multiple Analyte Identification

**DOI:** 10.1002/advs.202414688

**Published:** 2025-02-17

**Authors:** Woo Hyun Kim, Sungwoo Lee, Myeong Jin Jeon, Kwon Jun Lee, Jong‐Hak Park, Dae Won Park, Sungho Park, Sang Jun Sim

**Affiliations:** ^1^ Department of Chemical and Biological Engineering Korea University 145, Anam‐ro, Seongbuk‐gu Seoul 02841 Republic of Korea; ^2^ Department of Chemistry Sungkyunkwan University Suwon 16419 Republic of Korea; ^3^ Institute of Basic Science Sungkyunkwan University Suwon 16419 Republic of Korea; ^4^ Department of Emergency Medicine Korea University Ansan Hospital Korea University College of Medicine Ansan 15355 Republic of Korea; ^5^ Division of Infectious Diseases Department of Internal Medicine Korea University Ansan Hospital Korea University College of Medicine Ansan 15355 Republic of Korea; ^6^ Department of Chemistry Yonsei University Seoul 03722 Republic of Korea

**Keywords:** 3D bimetallic alloy nanoarchitecture, all‐in‐one multiplex detection, differential diagnosis of sepsis, soluble protein, surface‐enhanced Raman scattering (SERS)

## Abstract

Rapid and accurate differential diagnosis of infections, sepsis, and septic shock is essential for preventing unnecessary antibiotic overuse and improving the chance of patient survival. To address this, a 3D gold nanogranule decorated gold‐silver alloy nanopillar (AuNG@Au‐AgNP) based surface‐enhanced Raman scattering (SERS) biosensor is developed, capable of quantitatively profiling immune‐related soluble proteins (interleukin three receptor, alpha chain: CD123, programmed cell death ligand 1: PD‐L1, human leukocyte antigen–DR isotype: HLA‐DR, and chitotriosidase: ChiT) in serum samples. The 3D bimetallic nanoarchitecture, fabricated using anodized aluminum oxide (AAO), features a uniform structure with densely packed nanogaps on the heads of Au‐Ag alloy nanopillars, enabling fast, simple, and replicable production. The proposed biosensor achieves accurate results even with low detection limits (4–6 fM) and high signal consistency (relative standard deviation (RSD) = 1.79%) within a one‐step multi‐analytes identification chip with a directly loadable chamber. To enhance the diagnostic performance, a support vector machine (SVM) based machine learning algorithm is utilized, achieving 95.0% accuracy and 95.8% precision in classifying healthy controls, infections with and without sepsis, and septic shock. This advanced 3D plasmonic bimetallic alloy nanoarchitecture‐based SERS biosensor demonstrates clinical usefulness for sepsis diagnosis and severity assessment, providing timely and personalized treatment.

## Graphical abstract

The advanced 3D plasmonic bimetallic alloy nanoarchitecture‐based SERS biosensor is developed for differential diagnosis and severity prediction of sepsis, capable of detecting immune‐related serum proteins with high sensitivity (4–6 fM) and reproducibility. Combined with a machine learning algorithm achieving 95% accuracy, this outstanding biosensor enables effective clinical diagnosis of sepsis, supporting timely and personalized treatment.

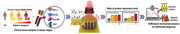

## 1 Introduction

As the global mean age increases, the demand for personalized diagnosis and treatment grows. Among these, the diagnosis of sepsis represents a critical healthcare challenge, affecting millions of people each year. According to the World Health Organization, in 2020, sepsis contributed to ≈11 million deaths globally, accounting for nearly 20% of all fatalities.^[^ ^1^ ^]^ The severity of sepsis is often intensified by infection due to weakened immune responses, making early and precise diagnosis is crucial for improving treatment outcomes.^[^ ^2^ ^]^ However, the existing diagnostic technologies for sepsis are limited, often leading to delayed interventions that significantly elevate mortality rates.

Current diagnostic approaches primarily focus on the detection of inflammation‐related biomarkers, such as C‐reactive protein (CRP)^[^ ^3^ ^]^ and procalcitonin (PCT),^[^ ^4^ ^]^ or rely on clinical evaluations such as the Sequential Organ Failure Assessment (SOFA) score.^[^ ^5^ ^]^ These methods are generally effective for assessing a patient's condition however they require considerable time and lack precision for early‐stage sepsis diagnosis.^[^ ^6^ ^]^ Although essential for identifying the causative pathogens, gold‐standard microbial culture techniques are time‐consuming and frequently result in false negatives owing to the absence of pathogens in the bloodstream at the time of testing.^[^ ^7^, ^8^ ^]^

The limitations of conventional diagnostic methods necessitate the empirical administration of broad‐spectrum antibiotics for sepsis, which may be ineffective for some patients.^[^ ^9^ ^.^ ^10^ ^]^ Moreover, the altered pharmacokinetics observed during sepsis progression further compromise the effectiveness of treatment.^[^ ^11^ ^]^ Thus, rapid, sensitive, and cost‐effective diagnostic tools capable of identifying key biomarkers associated with both the early onset and progression of sepsis are urgently needed.

Recent studies have focused on identifying novel soluble proteins in the bloodstream that can serve as accurate indicators of sepsis.^[^ ^12^ ^]^ Biomarkers such as soluble programmed death ligand‐1 (sPD‐L1),^[^ ^13^, ^14^ ^]^ interleukin‐3 receptor alpha chain (CD123),^[^ ^15^ ^]^ human leukocyte antigen–DR isotype (HLA‐DR)^[^ ^16^, ^17^ ^]^ and chitotriosidase (ChiT)^[^ ^18^ ^]^ have demonstrated the potential to provide insights into immune responses during sepsis. These biomarkers exhibit distinct expression patterns that can aid in the differential diagnosis and accurate identification of various stages of sepsis, thereby enabling more personalized treatment strategies.

Nanoplasmonic biosensors based on surface‐enhanced Raman scattering (SERS) have emerged as promising technologies for detecting these soluble proteins with high sensitivity.^[^ ^19^ ^]^ SERS enables the amplification of Raman signals by utilizing metal nanostructures, that generate localized surface plasmon resonances.^[^ ^20^, ^21^, ^22^ ^]^ The ability of SERS‐based biosensors to provide detailed molecular fingerprints, combined with their potential for multiplex detection, makes them a valuable tool for clinical diagnostics.^[^ ^23^, ^24^ ^]^ However, traditional SERS platforms are often limited by background noise in complex biological samples, hindering their sensitivity and reliability.^[^ ^25^ ^]^

To overcome these challenges, the development of 3D plasmonic bimetallic alloy nanostructures offers significant advantage. These nanostructures, comprising gold‐silver alloy nanopillars, exhibited superior SERS effects due to the synergistic properties of both metals.^[^ ^26^, ^27^ ^]^ Gold (Au) provides oxidation resistance, whereas silver (Ag) enhances plasmonic activity, resulting in high detection sensitivity and signal reproducibility. Furthermore, using the Au sputtering technique, Au nanogranules are deposited onto the Au‐Ag alloy nanopillars, creating rough surfaces with high surface curvatures. This process results in a dense array of SERS‐active sites, commonly known as electromagnetic “hot spots”.^[^ ^25^, ^28^ ^]^ These combined factors facilitate the detection of biomarkers, even at extremely low concentrations, allowing for real‐time monitoring of sepsis progression and severity.

This study proposes a novel SERS biosensor platform utilizing a 3D Au nanogranule decorated Au‐Ag alloy nanopillar (AuNG@Au‐AgNP) nanoarchitecture to detect sepsis‐associated soluble protein biomarkers in blood samples. This cutting‐edge platform demonstrated high sensitivity and selectivity, with the potential to distinguish between various stages of sepsis. By incorporating machine learning algorithms, we further improved diagnostic accuracy, offering a robust tool for the early detection and personalized treatment of sepsis. The study highlights the significant potential of SERS‐based nanoplasmonic biosensors for advancing sepsis diagnostics at various stages and ultimately improving the patient outcomes.

## 2 Results and Discussion

### 2.1 Preparation and Characterization of Advanced 3D Plasmonic Bimetallic Alloy Nanoarchitecture‐Based SERS Biosensor

**Figure 1:**
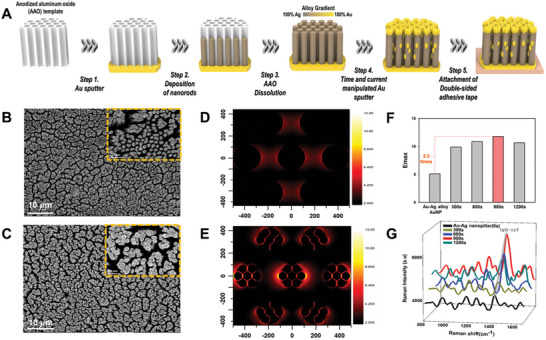
Fabrication of the 3D AuNG@Au‐AgNP Substrate. A) Scheme of the fabrication process. B, C) SEM images showing the top view of (B) the Au‐Ag NPs and (C) the fabricated 3D AuNG@Au‐AgNP SERS substrates after metal sputtering. D, E) Numerical simulations depicting the electromagnetic near‐field distribution for (D) Au‐Ag NPs without Au nanogranules and (E) the 3D AuNG@Au‐AgNP SERS substrates. F) Simulated maximum SERS enhancement factor, proportional to the fourth power of the electromagnetic field magnitude (|E|^4^). G) SERS spectra of IgG‐cyanine 3 and SERS signal intensity at 1321 cm^−1^ (characteristic of Cy3) for 3D AuNG@Au‐AgNP substrates with varying Au nanogranule sizes, using 10 nM IgG‐cyanine 3 as a model analyte.

Ensuring high sensitivity and selectivity for the clinically relevant detection of target soluble proteins via SERS requires the fabrication of 3D plasmonic nanostructures with high density plasmonic hotspots to generate strong near‐field amplification. To address this issue, we adopted porous anodized aluminum oxide (AAO) templates possessing honeycomb porous structures which allowed the synthesis of 2D hexagonally packed arrays of nanopillars through potentiostatic electrochemical deposition (**Figure** **1**A). First, one side of the AAO template was coated with a thin Au layer via sputtering to make the AAO template conductive. Then, Au‐Ag alloy nanopillars (Au‐Ag NPs) were synthesized in porous AAO templates by depositing a plating solution containing Au (210 mM) and Ag (900 mM) precursors. The alloy composition of the nanorods was manipulated by adjusting the Au/Ag ion ratio in the mixture. Additionally, the length and diameter of the nanorods were controlled by tuning the total charge passed during synthesis. Subsequently, the AAO template was removed by the addition of 3 M NaOH solution dissolving that template. To further enhance the SERS signal, Au nanogranules were deposited on top of the nanopillars via sputtering. Au nanogranules were predominantly deposited on the top surface of the Au‐Ag NPs, providing high surface energy. Finally, the Au‐Ag NPs were attached to adhesive tape for further analysis.

The field‐emission scanning electron microscopy (FE‐SEM) image represented that the Au‐Ag NPs were homogeneously synthesized over a large area (Figure 1B). The nanorods had an average diameter of 264 ± 34 nm, a total length of 1152 ± 90 nm, and a density of 8.1 ± 0.3 ea µm^−2^. After Au sputtering, Au nanogranules were preferentially formed on top of the Au‐Ag NPs (Figure 1C). Energy dispersive spectroscopy (EDS) analysis revealed that the percentages of Au and Ag in the Au‐Ag NPs were 88.31% and 11.69% by weight, respectively (Figure S1A, B, Supporting Information). The percentage of Au increased to 92.21%, while that of Ag decreased to 7.9% after Au sputtering (Figure S1C, D, Supporting Information).

We optimized the elemental composition of the Au‐Ag NPs by finite‐difference time‐domain (FDTD) analysis to achieve the highest electric field enhancement for SERS analysis (Figure S2, Supporting Information). The variation of Au:Ag ratio of Au‐Ag NPs from 1:9 to 9:1 (the elemental composition of Au and Ag of Au‐Ag NPs), including pure Au and Ag nanopillars were tested. From the FDTD simulation, the highest electric field enhancement was observed for the Au:Ag ratio of 9:1 (Emax = 5.08) among the comparisons. Further investigation of the electromagnetic near‐field focusing capabilities of nanopillars with varying elemental compositions was performed using SERS with IgG‐cyanine 3 as the analyte (Figure S3A, Supporting Information, laser wavelength: 785 nm, laser power: 10.67 mW). IgG‐cyanine 3 analytes exhibited multiple SERS peaks, with the strongest signal observed at 1321 cm^−1^. For the analysis of the peaks, 1321 cm^−1^ was then used based on this observation. As predicted from the FDTD simulation, an Au:Ag ratio of 9:1 showed the highest SERS signal. Although the FDTD simulation predicted a high near‐field focusing capability (Emax = 5.08) for the Au‐Ag NPs with an Au:Ag ratio of 1:9, the SERS signal was weak due to rapid Ag oxidation (Figure S3B, Supporting Information). Au‐Ag alloy nanostructures, combining the high stability of Au with the large extinction cross‐section of Ag, exhibited high SERS enhancement as expected. As the Ag composition increased beyond an Au ratio of 9:1, the plasmonic bands of the Au‐Ag NPs began to deviate from the excitation wavelength (785 nm), resulting in decreased SERS enhancement. However, starting from an Au ratio of 5:5, further increases in Ag content led to improved SERS enhancement.

Achieving highly sensitive detection of low‐abundance proteins in biological samples, a hierarchical structure was developed to generate additional SERS enhancement. By controlling the Au sputtering time (from 300 to 1200 s) while maintaining the current at 30 mA, various diameters of AuNG@Au‐AgNP from 211 ± 33 nm to 364 ± 64 nm (Figure S4, Supporting Information) were tested as well. We investigated the side sections of AuNG@Au‐AgNPs by cutting the substrate with an ion‐beam cross‐section polisher. The SEM images of the side sections of AuNG@Au‐AgNP and Ag‐AuNP showed that Au nanogranules were predominantly decorated on the heads of the nanopillars (Figure S5, Supporting Information).

To compare the electric‐field enhancement, FDTD simulations were conducted using four different Au nanogranule sizes (211, 244, 323, and 364 nm, measured from the SEM images). This numerical simulation results indicated that the electric field enhancement reached a maximum of 11.76 when the size of the Au nanogranules was 323 ± 27 nm. Furthermore, the simulations predicted that this configuration would provide a 2.3 times more intense SERS amplification effect than the Au‐Ag NPs (Figure 1D,E,F; Figure S6, Supporting Information). This trend can be ascribed to the surface curvature of the Au nanogranules formed in the head region. As the size of the nanogranules increases, the curvature of the surface correspondingly diminishes. This reduction in curvature lowers the absorption and inelastic scattering of light at the surface, leading to a weakened surface electromagnetic field and, consequently, a decline in the overall SERS intensity.^[^ ^28^, ^29^ ^]^ In contrast, the gaps between Au nanogranules were too small to accomplish efficient SERS enhancement under the conditions of 300 and 600 s, resulting in inadequate near‐field amplification induced by plasmonic coupling.^[^ ^30^, ^31^ ^]^ From the SERS measurement of Au‐Ag NPs and the Au nanogranule decorated Au‐Ag alloy nanopillar, the SERS signal from IgG‐cyanine 3 at 1321 cm^−1^ was the highest, showing the highest near‐field focusing capabilities (Figure 1G; Figure S7, Supporting Information). This aligns with our prediction from the FDTD simulation when the Au nanogranule size was 323 ± 27 nm. These results demonstrate that the 3D plasmonic bimetallic nanoarchitecture generates intense SERS signals, making it promising for detecting target biomarkers, including low‐concentration soluble proteins in biofluids.

The efficient analysis of various immune response‐related proteins expression is crucial for the accurate diagnosis of sepsis and severity prediction. Thus, we constructed a SERS biosensor based on 3D AuNG@Au‐AgNPs and 3D printed multiplex chip with directly loadable chamber for the simultaneous detection of soluble proteins in the blood. A 1 mL sample of whole blood from a 3 mL EDTA tube was transferred to a 1.5 mL protein LoBind tube, and a 3D‐printed multiplex chip was attached to hold multiple 3D AuNG@Au‐AgNP nanostructure‐based substrates. The blood samples were then centrifuged for 10 min using a benchtop centrifuge. The length of the 3D‐printed multiplex chip was optimized to allow incubation only within the separated plasma and serum layers. In addition, the SERS signal measurements can be performed within a few seconds. This advanced SERS system enables rapid and simple plasma and serum separation while allowing the simultaneous incubation of multiple substrates.

**Figure 2:**
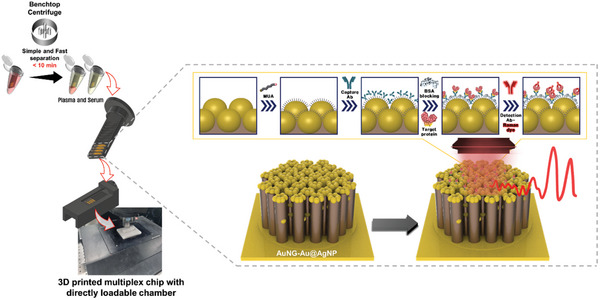
Schematic of soluble protein detection in blood using the 3D AuNG@Au‐AgNP based SERS biosensor with the 3D‐printed multiplex chip and direct‐loadable chamber. This biosensor allows direct sample incubation using an antigen‐antibody sandwich assay to detect target soluble proteins. Blood and serum samples were centrifuged with a benchtop centrifuge for 10 min. The multiplex chip containing four 3D AuNG@Au‐AgNP substrates attached with double‐sided tape was incubated during centrifugation. Following incubation, SERS signals were measured using the 3D‐printed direct‐loadable chamber.

**Figure** **2** illustrates the configuration of the 3D AuNG@Au‐AgNP based SERS biosensor, designed for the simultaneous identification of four soluble proteins relevant to the differential diagnosis and severity assessment of sepsis in serum. To ensure specific detection of the four target soluble proteins, a sandwich assay was implemented using capture and detection antibodies that recognize the distinct motifs of each target soluble protein.^[^ ^32^, ^33^ ^]^ A self‐assembly monolayer of 11‐mercaptoundecanoic acid (MUA) was used to immobilize the capturing antibodies onto Au nanogranules. Figure S8 (Supporting Information) shows that the thiol groups of 11‐MUA formed Au‐S bonds on the Au nanogranule surface, whereas the carboxyl groups enabled crosslinking with antibody amines via an N‐hydroxysuccinimide (NHS)‐1‐Ethyl‐3‐diaminopropyl carbodiimide (EDC) reaction, facilitating the attachment of capture antibodies utilizing NH_2_ as confirmed by the C ═ O, N‐H, and CH_2_ peaks in the FT‐IR spectrum.^[^ ^34^ ^]^ Bovine serum albumin (BSA) was used as a surface blocker to maintain antibody integrity and prevent non‐specific interactions.^[^ ^35^ ^]^ This pretreatment step played a critical role in ensuring that the analytical results accurately reflect the presence of the target soluble proteins. Each detection antibody was conjugated with Raman reporters (cyanine 3: Cy3, cyanine 5: Cy5, cyanine 7: Cy7, and fluorescein isothiocyanate: FITC) specific to the corresponding target protein, enabling multiplex detection within a 3D‐printed single chip (Figure S9, Supporting Information). Additionally, as described in Figures S9 and S10 (Supporting Information), a 3D‐printed chamber was fabricated to enable the direct loading of the chip into the SERS measurement device, facilitating rapid analysis compared to conventional methods. These results suggested that the SERS sensor could effectively and efficiently identify target blood‐based protein biomarkers.

### 2.2 Evaluation of Soluble Protein Analytical Performance of Advanced 3D Plasmonic Bimetallic Alloy Nanoarchitecture‐Based SERS Biosensor

While SERS sensitivity has advanced over time, its empirical application for analyte quantification in the clinical field has been limited due to poor reproducibility and uniformity.^[^ ^36^ ^]^ Thus, to overcome these issues, we evaluated our 3D SERS biosensor featuring 3D bimetallic alloy nanopillar with Au nanogranule for its ability to quantify four sepsis related soluble immune proteins using Raman reporters (CD123‐Cy7 peak at 1131 cm⁻¹, PD‐L1‐Cy3 peak at 1240 cm⁻¹, ChiT‐FITC peak at 1516 cm⁻¹, and HLA‐DR‐Cy5 peak at 1321 cm⁻¹).^[^ ^37^, ^38^, ^39^, ^40^, ^41^ ^]^

**Figure 3:**
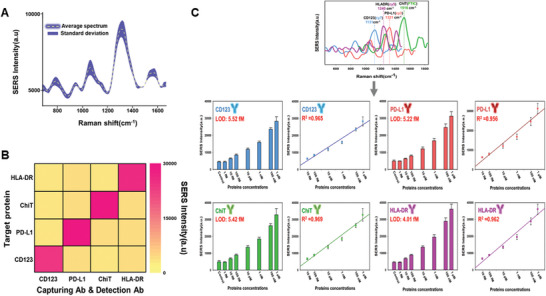
Evaluation of sensing performance of 3D AuNG@Au‐AgNP‐based SERS biosensor A) Averaged SERS spectra (dashed line) with standard deviations (shaded area) for PD‐L1 (Cy3) at 100 nM, measured across a 50 × 50 µm area (2500 points) on a single SERS biosensor. B) Selectivity assessment of the 3D bimetallic alloy nanoarchitecture‐based SERS biosensor through SERS intensity measurements at specific Raman reporter peaks, validated for target soluble proteins (CD123, PD‐L1, ChiT, HLA‐DR) using a confusion matrix. C) Sensitivity analysis of the 3D AuNG@Au‐AgNP‐based SERS biosensor: SERS spectra over a concentration gradient and corresponding plot of specific peak intensities (1131 cm⁻¹, 1240 cm⁻¹, 1321 cm⁻¹, and 1516 cm⁻¹ for CD123, HLA‐DR, PD‐L1, and ChiT, respectively) against soluble protein concentrations in spiked human serum. Linear regression analysis was performed to assess the correlation between soluble protein concentration and specific peak intensity. Error bars represent standard deviations from 30 measurements.

As illustrated in **Figure** **3** **A**, the 3D plasmonic bimetallic alloy SERS biosensor demonstrated a mean spectrum with a highly consistent standard deviation (SD) across 2500 points within the 50 × 50 µm mapping area of the substrate, using a 1 µm diameter laser spot, at a PD‐L1 concentration of 100 nM. Moreover, to further evaluate signal reproducibility, the relative standard deviation (RSD) of the 1321 cm^−1^ SERS peak was measured at 30 randomly selected points using PD‐L1 (Cy3) at a concentration of 100 nM, yielding an RSD of 1.79% (Figure S11, Supporting Information). The 3D plasmonic bimetallic alloy nanoarchitecture‐based SERS biosensor exhibited highly consistent signal intensities, ensuring reliable reproducibility. The high reproducibility of the SERS signals in our system was attributed to the uniform size and well‐separated positioning of the nanopillars, preventing them from clustering together. In typical SERS setups, achieving a homogeneous nanogap across a large area is challenging, especially for assemblies such as Au nanospheres, which form a 0D nanogap (point‐to‐point contact).^[^ ^42^ ^]^ However, these nanogaps are difficult to fabricate. Using a hexagonally packed porous AAO template, we achieved well‐spaced nanopillars that provided a reproducible electromagnetic near‐field, resulting in a homogeneous and reliable SERS substrate and consequently enabling the quantification of target proteins.^[^ ^43^ ^]^

In blood‐based biosensor systems, managing cross‐reactivity signals from competing analytes is essential, as it can compromise the detection accuracy and reliability.^[^ ^44^ ^]^ To assess the selectivity of the developed SERS biosensor, cross‐reactivity tests were conducted using serum samples spiked with the four target soluble proteins. A strong SERS signal intensity was observed in the presence of the target proteins. In contrast, no significant SERS signal was observed for non‐target proteins, even at concentrations 100 times higher than those of the target proteins (Figure 3B). This high selectivity is due to two primary factors, nanometer‐sized structures that minimize non‐specific binding and bovine serum albumin (BSA), which blocks the unoccupied AuNG‐Au@AgNP surface and prevents non‐specific adsorption.^[^ ^35^ ^]^ Furthermore, the recovery test results from plasma spiked with various soluble proteins ranged from 96.0% to 110.0% (Table S1, Supporting Information). These data highlight that the advanced SERS biosensor accurately identified target soluble proteins in blood without interference, establishing it as a reliable tool for diagnosing and predicting sepsis.

The quantification of immune‐related soluble protein biomarkers in the blood is essential for the accurate differential diagnosis of sepsis because of their expression patterns throughout the disease.^[^ ^3^, ^45^ ^]^ These biomarkers are closely associated with the early hyperinflammatory phase and subsequent compensatory anti‐inflammatory response.^[^ ^46^ ^]^ However, the concentration of soluble proteins in the blood is remarkably low, and numerous interfering substances pose challenges for accurate quantitative profiling. Moreover, conventional immunoassays, such as enzyme‐linked immunosorbent assay (ELISA), lack the sensitivity to detect trace amounts of these proteins in the blood. Therefore, a highly sensitive system is required. The sensitivity of the AuNG‐Au@AgNP‐based SERS biosensor was evaluated by measuring the SERS spectra of the target soluble proteins spiked in human serum samples at various concentrations. Using this analysis, the detection limits and dynamic ranges of the SERS biosensor were determined. As shown in Figure 3C, we identified spectra containing distinct Raman reporter peaks corresponding to each soluble target protein. Additionally, a strong linear correlation between SERS intensity and protein concentration over a logarithmic range from 10 fM to 1 µM was confirmed, encompassing physiological levels in human plasma (R^2^ > 0.950) (Figure 3C).^[^ ^14^, ^15^, ^16^, ^17^ ^]^ To establish a quantitative correlation between protein concentration and SERS intensity, we plotted a linear regression by fitting the Raman reporter peak intensity to the target protein concentration, as shown in Figure 3C. The linear regression equations were obtained as follows:

(1) $$y=2638\log x+2533,\;R^{2}=0.965\;\mathrm{for}\;\mathrm{CD}123$$

(2) $$y=2974\log x+1623,\;R^{2}=0.956\;\mathrm{for}\;\mathrm{PD}-L1$$

(3) $$y=3109\log x+2261,\;R^{2}=0.969\;\mathrm{for}\;\mathrm{ChiT},\;\mathrm{and}$$

(4) $$y=3532\log x+1271,\;R^{2}=0.962\;\mathrm{for}\;\mathrm{HLA}-\mathrm{DR}$$

where *x* is the protein concentration.

The limits of detection (LOD), calculated using the linear regression equations described in the Materials and Methods section, were 5.52, 5.22, 5.42, and 4.01 fM for CD123, PD‐L1, ChiT, and HLA‐DR, respectively. The detection limit for the target soluble proteins was < 10 fM. Compared to previously reported SERS biosensors^[^ ^47^, ^48^ ^]^ and other amplification methods,^[^ ^49^, ^50^, ^51^, ^52^ ^]^ such as fluorescent or electrochemical biosensors, the 3D plasmonic bimetallic alloy nanoarchitecture‐based SERS biosensor demonstrated significantly superior sensitivity. These detection limits were at least six times lower than that of other SERS‐based biosensors and 132 times lower than that of other types of biosensors (Table S2, Supporting Information). This outstanding sensitivity was attributed to plasmonic coupling within the densely packed 3D nanogaps of the AuNG@Au‐AgNP structures, resulting in abundant 3D SERS hotspots.^[^ ^53^, ^54^ ^]^ Considering the low abundance of soluble proteins in blood and the significant differences in their expression patterns during sepsis progression,^[^ ^55^, ^56^ ^]^ our SERS biosensor, with its ultra‐low detection limits and broad dynamic range, is expected to be a valuable tool for analyzing soluble protein expression patterns in blood for diagnostic applications.

### 2.3 Dual Track Detection Approaches of Target Soluble Proteins in Human Serum Samples for Assessing the Clinical Significance of 3D Plasmonic Bimetallic Alloy Nanoarchitecture‐Based SERS Biosensor

Recent studies have emphasized the need for personalized treatment strategies for patients with infections, both with and without sepsis, as well as those with septic shock.^[^ ^57^ ^]^ The classification of these clinical groups mainly depends on physician's clinical judgment of the patient.^[^ ^58^ ^]^ Thus, accurate differentiation between normal, infected without sepsis, sepsis, and septic shock patients is crucial to ensure appropriate antibiotic use and clinical care.^[^ ^59^, ^60^ ^]^

**Figure 4:**
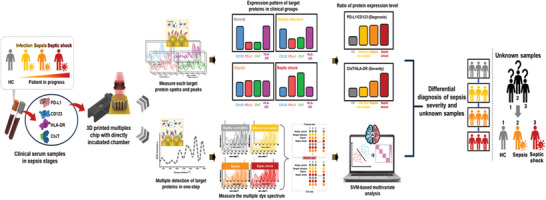
Clinical significance evaluation using the 3D bimetallic alloy nanoarchitecture‐based SERS biosensor and dual track detection approaches with machine learning. Schematic illustration of dual track detection approaches for differential diagnosis among clinical groups, utilizing expression level ratios and SVM algorithm analysis, with all‐in‐one incubation for complex SERS spectral measurement enabling multiple analyte identification.

To confirm the clinical applicability of differentiating between normal, infected (with and without sepsis), and septic shock, clinical serum samples from 10 individuals per group (detailed demographic information for all samples is provided in Table S3, Supporting Information) were prepared. Dual‐track detection approaches have been applied to clinical serum sample analysis for sepsis diagnosis and severity prediction. SERS spectra and analysis of the signal intensity of the peaks corresponding to each target soluble protein enabled simultaneous multiplex quantitative profiling of target protein expression levels in human serum. As shown in **Figure** **4**, two analytical approaches were employed. In the first approach, we analyzed the expression patterns of each target soluble protein using different substrates and conducted quantitative measurements based on the signal intensities from the Raman reporter peaks. The PD‐L1/CD123 ratio was used as a diagnostic marker, whereas the ChiT/HLA‐DR ratio served as an indicator of sepsis severity, enabling the differential diagnosis of healthy controls (HC), infection with and without sepsis, and septic shock.

**Figure 5:**
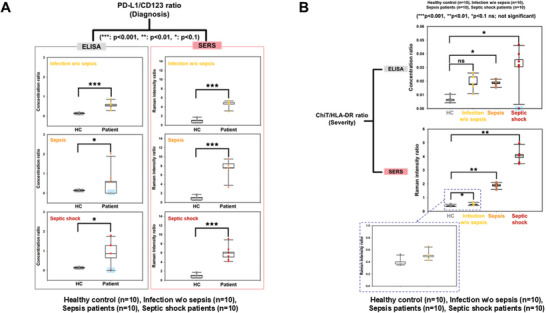
Clinical significance evaluation for differential diagnosis among clinical groups utilizing expression level ratios using the 3D bimetallic alloy nanoarchitecture‐based SERS biosensor. Box plots showing the expression level ratios of A) PD‐L1/CD123 and B) ChiT/HLA‐DR, comparing healthy controls and symptomatic groups (*n* = 10 per group) for sepsis diagnosis and severity prediction, samples undetected by ELISA represented in skyblue rectangles (***p < 0.001, **p < 0.01, *p < 0.1 ns; not significant).

Above all, the expression patterns of the target soluble proteins, measured through SERS peak intensity, demonstrated that the PD‐L1/CD123 ratio differentiated the three patient groups from the HC group, although it failed to provide a distinct separation between them (**Figure** **5A**). Comparison with data obtained from the conventional ELISA, using the Mann‐Whitney U test for p‐value confirmation between the HC and symptomatic groups, validated the results.^[^ ^61^, ^62^ ^]^ The ELISA results showed lower clinical significance (*p* < 0.01) in the sepsis and septic shock groups, with some samples yielding no measurable results. In contrast, our SERS biosensor demonstrated a higher clinical significance (*p* < 0.001) across all three patient groups. These findings suggest that the 3D plasmonic bimetallic alloy nanoarchitecture‐based SERS biosensor offers more reliable differentiation between healthy subjects and the three patient groups than ELISA.

An analysis of the ChiT/HLA‐DR ratio demonstrated significant differences between the healthy and three patient groups. As shown in Figure 5B, the proposed SERS biosensor successfully classified sepsis and septic shock (*p* < 0.01), except for differentiating between HC and infections without sepsis (*p* < 0.1). In contrast, ELISA showed no clinically significant differences among the clinical groups (*p* < 0.1 and not significant). Hence, these results indicate that the developed SERS biosensor offers better clinical reliability than ELISA for distinguishing sepsis severity.

**Figure 6:**
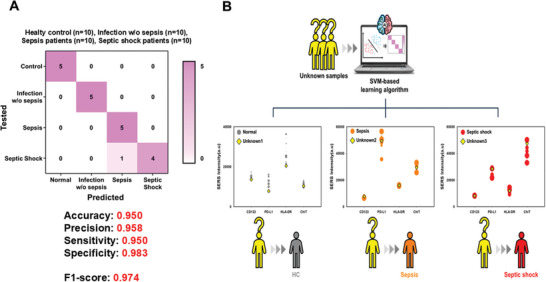
A) Confusion matrix displaying the diagnostic accuracy of SVM classifiers in distinguishing healthy control group (*n* = 10) from symptomatic groups (*n* = 30). B) Differential diagnosis of clinical unknown patients (*n* = 3) using SVM algorithm‐based machine learning.

While previous analytical methods have demonstrated success in diagnosing and predicting the severity of sepsis and septic shock compared to ELISA, this approach faces challenges in achieving clinically significant differentiation for the simple infection group. Additionally, the previous approach resulted in poor efficiency of time and cost, measuring four substrates with a different Raman reporter individually. To overcome these limitations, we implemented an all‐in‐one incubation process on a single substrate with four target proteins, enabling the simultaneous measurement of complex SERS spectra for multiple target soluble proteins to diminish the measurement process and time. Evaluating the effectiveness of the machine learning‐based analysis model in our biosensor system, the three most widely used classification algorithms (namely, linear discriminant analysis, LDA; k‐nearest neighbors, KNN; and support vector machines, SVM) were tested using the Raman peak data measured by our sensor.^[^ ^63^, ^64^ ^]^ Figure S12 (Supporting Information) shows the confusion matrices of each model used to distinguish between the patient and control groups. The results revealed that all three methods showed significant discrimination accuracy and F1‐score.^[^ ^65^ ^]^ In particular, SVM exhibited the most accurate diagnostic results, with an accuracy of 95% and an F1‐score of 0.974. **Figure** **6**A compares the diagnostic performance indices (accuracy, precision, sensitivity, and specificity), with the SVM‐based differentiation showing 95.8% precision, 95.0% sensitivity, and 98.3% specificity. Instead of relying on SERS intensity values from specific reporter peaks, this method analyzes complex spectral data in one step, enabling clinically significant classification across the four clinical groups and improving the identification of multiple analytes.^[^ ^66^, ^67^ ^]^ The diagnostic power of the integrated system was further validated by an additional blind sample analysis. The SERS signals from three clinical samples from unknown patient groups were measured using our biosensor and analyzed from the established SVM model (Figure 6B). The results showed that the diagnostic system could correctly classify the three samples using SERS measurements, implying that our integrated system is capable of differentially diagnosing various stages of sepsis.

## 3 Conclusion

In this study, we successfully developed a highly sensitive and selective 3D bimetallic alloy nanoarchitecture‐based SERS biosensor that can detect sepsis‐associated soluble proteins in blood samples. Using an advanced nanoplasmonic design, our biosensor can differentiate between various stages of sepsis, especially infections with and without sepsis, offering a substantial improvement in diagnostic accuracy. The incorporation of machine learning algorithms further enhanced the diagnostic precision of the platform, underscoring its potential for early sepsis detection and enabling a more personalized approach to patient treatment. This study emphasizes the transformative impact of SERS biosensors on sepsis diagnostics, advancing toward faster, more accurate, and patient‐specific interventions. The findings of this study highlight the potential of this biosensor in improving patient outcomes in the clinical field and present a promising avenue for future research on nanotechnology‐driven healthcare applications.

## 4 Experimental Section

### Reagents and Materials

The AAO template was sourced from Whatman. The Ag plating solution (1025 RTU) and Au plating solution (Orotemp 24 RTU) were obtained from Technic Inc. Nitric acid (HNO₃, 60%) and sodium hydroxide (NaOH, 98%) were purchased from Samchun (Korea). Protein LoBind tubes (1.5 mL) were purchased from Eppendorf Korea. Chemicals including 11‐mercaptoundecanoic acid (MUA), N‐hydroxysuccinimide (NHS), 1‐ethyl‐3‐diaminopropyl carbodiimide (EDC), and 2‐(N‐morpholino) ethanesulfonic acid (MES) buffer were acquired from Sigma Aldrich (Korea). Bovine serum albumin (BSA) (no. A7638) and human plasma (No. P9523) were obtained from Sigma‐Aldrich (Korea). A strongly adhesive PET substrate double‐sided tape was purchased from KGK Chemical Corporation. Human recombinant proteins CD123, PD‐L1, and ChiT were sourced from Sino Biological Inc. (China), and human recombinant HLA‐DR was obtained from Abcam (United Kingdom). PD‐L1 (6C8) and Cy3‐conjugated PD‐L1 (3B11) monoclonal antibodies were obtained from Bioss Antibodies (USA). Anti‐CD123 antibody, PE/Cy7 anti‐CD123 antibody [6H6], anti‐HLA‐DR antibody, and PE/Cy5 anti‐HLA‐DR antibody [LN3] were purchased from Abcam (United Kingdom). Mono and poly‐(FITC) chitotriosidase antibodies were acquired from LSBio (USA).

### Synthesis of 3D AuNG@Au‐AgNP Nanoarchitecture

We prepared nanoforests using an electrochemical deposition method with a three‐electrode system and commercial AAO templates from Whatman (≈270 nm in pore size and 25 mm in diameter). A thin layer of Au was evaporated onto one side of the AAO template using a sputter coater (Cressington Scientific Instruments) at a current of 30 mA for 600 s, which served as the working electrode. Subsequently, to synthesize the Au/Ag alloy nanopillar, we applied a mixture of Au plating solution (Orotemp 24 RTU, Technic Inc.) and Ag plating solution (1025 RTU, Technic Inc.) at ‐0.95 V (versus Ag/AgCl). The AAO template was dissolved using 3 M NaOH to release the Au‐Ag alloy nanopillars, which were then thoroughly rinsed with triple distilled water and dried in an oven to completely remove moisture. Finally, to selectively decorate the Au nanogranules onto the head of the Au‐Ag alloy nanopillar, we used a sputter coater at a current of 30 mA from 300 to 1200 s. SEM images of the AuNG@Au‐AgNP cross‐sections were obtained using ion‐beam cross‐section polisher. for analysis of the percentages of Au and Ag in the Au‐Ag NPs To determine the Au:Ag ratio of Au:Ag ratio of Au‐Ag NPs, the elemental composition of Au and Ag was simultaneously measured using EDS during FE‐SEM measurement process.

### Detection of Soluble Protein using the 3D AuNG@Au‐AgNP Nanoarchitecture‐Based SERS Biosensor

As described in Figure 2, 3D AuNG@Au‐AgNPs were produced. To completely conjugate the antibodies onto the nanostructure, we utilized a self‐assembled monolayer of 11‐MUA (10 mM) containing thiol groups for the surface modification of the carboxyl group. NHS and EDC were dissolved in MES buffer at 1 M, ensuring pH stability. NHS and EDC solutions (each 500 µL) were mixed and then incubated with the AuNG@Au‐AgNP substrate for 10 min, followed by rinsing with distilled water and blowing N_2_. To immobilize the capture antibody, 5 µL of a 1 µM solution was applied to the substrate and incubated by NHS‐EDC reaction onto Au nanogranules for 15 min. To reduce non‐specific binding, 5 µL of blocking solution (1 wt.% BSA in 0.1x PBS buffer) was applied and incubated for 15 min, followed by rinsing with 0.1x PBS buffer and blowing N_2_. Subsequently, the recombinant soluble protein was diluted according to a concentration gradient (from 1 fM to 1 µM), spiked into human serum for quantitative analysis, and clinical samples were used as collected. A 10 µL volume was applied and incubated for 15 min. After washing with PBS buffer at pH 7, 5 µL of detection antibody containing a Raman reporter (1 µM) was applied and incubated for 15 min, followed by another PBS wash and blowing N_2_ before Raman signal measurement.

For the direct whole blood measurements, the capture antibody step was performed as previously described. Subsequently, during the target protein incubation step, we fabricated a multiplex chip and directly loadable chamber using Anycubic Photon M3 Max 3D printer and standard resin (chip: 0.5 mL resin, chamber: 4 mL resin, single production running time: 2 h). The 3D AuNG@Au‐AgNP substrate was attached to the 3D‐printed multiplex chip using double‐sided tape and loaded into 1 mL of EDTA‐treated whole blood in a 1.5 mL LoBind tube. The samples were then centrifuged for 10 min and incubated for 15 min. The subsequent detection antibody treatment followed the same protocol as that for the spiked serum. The protein samples labeled with Raman reporters were analyzed using a custom‐built Raman microscope (Weve, Korea). A 785 nm excitation laser, directed through a TU Plan ELWD 100× air objective lens (numerical aperture 0.6, working distance 0.56 mm, Nikon, Japan) attached to an upright microscope (Eclipse Ni‐U, Nikon), provided the excitation. The laser power was calibrated to 10.67 mW by using a PM100 power meter (Thorlabs, USA). Each sample underwent 0.5 s laser exposure with 20 accumulations, using a confocal slit size of 120 µm and an ND filter set at 32%. A FEX spectrograph (Weve, Korea), coupled to a Newton 920 CCD camera (Andor Technology, UK), captured the Raman signals. Raman mapping covered a 50 × 50 µm^2^ area with a step size of 1 µm. Data were processed using the RAON‐Vu software (Weve), Sigmaplot 10 (Systat Software Inc., CA, USA), and Origin 8 (OriginLab, MA, USA). The LOD was calculated using the formula LOD=3.3×δ/m, where σ denotes the standard deviation of the blank and m is the slope of the calibration curve.^[^ ^68^ ^]^

### Numeric Simulation of 3D AuNG@Au‐AgNP Nanoarchitecture

Numerical modeling and simulation of the nanostructure were performed using the Lumerical FDTD Solutions software (Lumerical Inc.). The structural dimensions were calculated using the GeoGebra graphic calculator. The simulations, based on SEM measurements of the Au‐Ag nanopillars (diameter: 264 nm, height: 1150 nm, and inter‐pillar spacing: 200 nm), were used to evaluate the electromagnetic field enhancement across various Au‐Ag alloy ratios (from 1:9 to 9:1) and with Au nanogranules of sizes 211, 244, 323, and 364 nm. A plane‐polarized light source with a wavelength of 785 nm was directed onto the nanostructure. The simulation mesh size was set at 1 nm, with an incident light frequency of 3.82 × 10^14^ Hz.^[^ ^69^ ^]^

### Preparation of Target Soluble Proteins for Sensing Performance Validation

Preparation of target soluble proteins for sensing performance validation: Four types of immune‐related proteins (CD123, PD‐L1, ChiT, and HLA‐DR) were obtained from ELISA kits (UK Antibodies.com), aliquoted into protein LoBind tubes, and stored at ‐80 °C for further use. Each biomarker was serially diluted in PBS buffer (10 mM, pH 7.4) to prepare stock solutions at concentrations ranging from 1 µM to 1 fM. The prepared biomarkers were then spiked into human serum to measure the optical signals at different biomarker concentrations.

### Recovery Test of Target Soluble Proteins

For the recovery test, human serum was spiked with target soluble proteins to prepare samples of the four target soluble proteins at a concentration of 10 pM. Following the detection of target soluble protein using the SERS biosensor, the SERS signal intensity was measured to determine the recovery rate.

### Clinical Serum Samples and Statistical Mann‐Whitney U Test

Human serum samples were collected from participants using serum separation tubes (SST) equipped with anticoagulant and separation gels. Following centrifugation at 5000 rpm for 30 min, the supernatant was carefully collected and promptly stored at ‐80 °C. The study included ten subjects per clinical group, including those diagnosed with infections with and without sepsis, those with septic shock, and healthy controls. All clinical samples utilized in this study were obtained from the Korea University Ansan Hospital with informed consent from each participant or their legal representative. This study was approved by the Ethics Committee and Institutional Review Board (IRB#2024AS0030). Ten microliters of each clinical sample were used for analysis in every experiment. Additionally, 30 optical signals were measured and averaged to obtain reliable results. Statistical significance was evaluated using the Mann‐Whitney U test,^[^ ^70^ ^]^ and the p‐value was calculated using Sigmaplot 10 (Systat Software Inc., CA, USA).

### Machine Learning‐Based Analysis

Patient classification was further conducted using machine learning algorithms. The training data were constructed using 10 healthy controls, 10 infected patients without sepsis, 10 patients with sepsis, and 10 patients with septic shock, with four characteristic Raman peaks corresponding to CD123, PD‐L1, HLA‐DR, and ChiT. Three classification methods were developed and utilized in this study: LDA, KNN, and SVM. To assess the performance of each classification model, the major measures of diagnostic power (confusion matrix, accuracy, F1 score, precision, sensitivity, and specificity) were compared.

## Conflict of Interest

The authors declare no conflict of interest.

## Supporting information



Supporting Information

## Data Availability

Research data are not shared.
