# Supplementary material for: Rapid and Differential Diagnosis of Sepsis Stages Using an Advanced 3D Plasmonic Bimetallic Alloy Nanoarchitecture‐Based SERS Biosensor Combined with Machine Learning for Multiple Analyte Identification
Source: Adv Sci (Weinh). 2025 Feb 17;12(14):2414688. doi: 10.1002/advs.202414688 (PMC11984904; doi:10.1002/advs.202414688)
Supplement: Supplementary file 1 — Supporting Information [file ADVS-12-2414688-s001.docx]

Supporting Information

Rapid and Differential Diagnosis of Sepsis Stages using an Advanced Three-Dimensional Plasmonic Bimetallic Alloy Nanoarchitecture-Based SERS Biosensor Combined with Machine Learning for Multiple Analyte Identification

Woo Hyun Kim, Sungwoo Lee, Myeong Jin Jeon, Kwon Jun Lee, Jong-Hak Park, Dae Won Park, Sungho Park* and Sang Jun Sim*


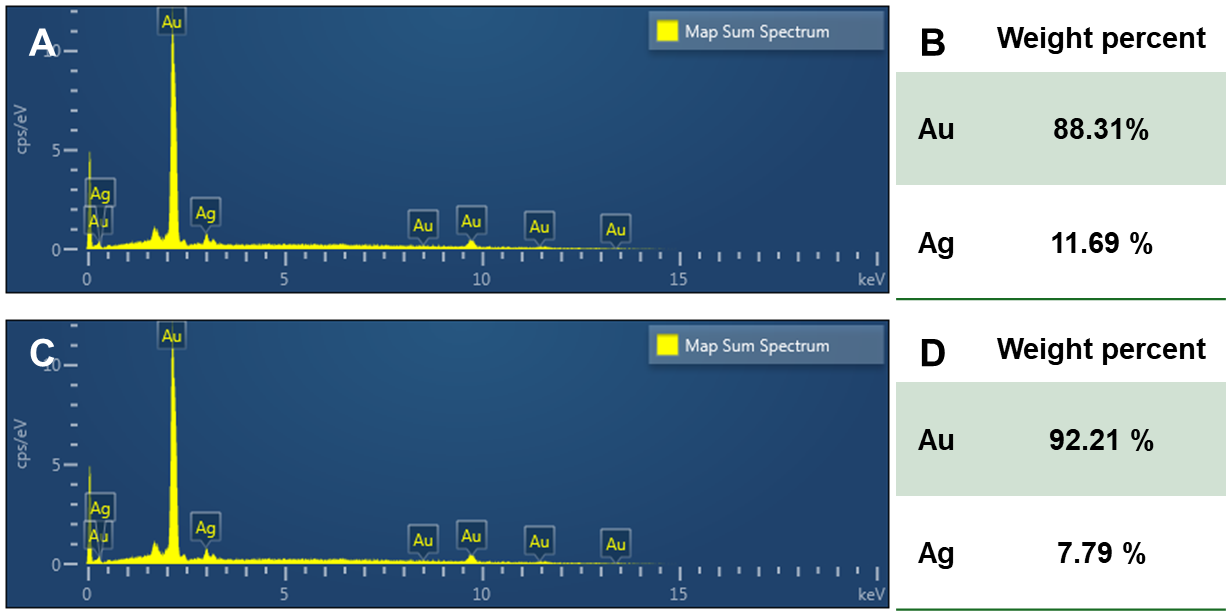


Figure S1. EDS spectrum and elemental composition of Au-Ag alloy nanopillar (A, B) and

Au-Ag alloy with Au nanogranule (C, D).


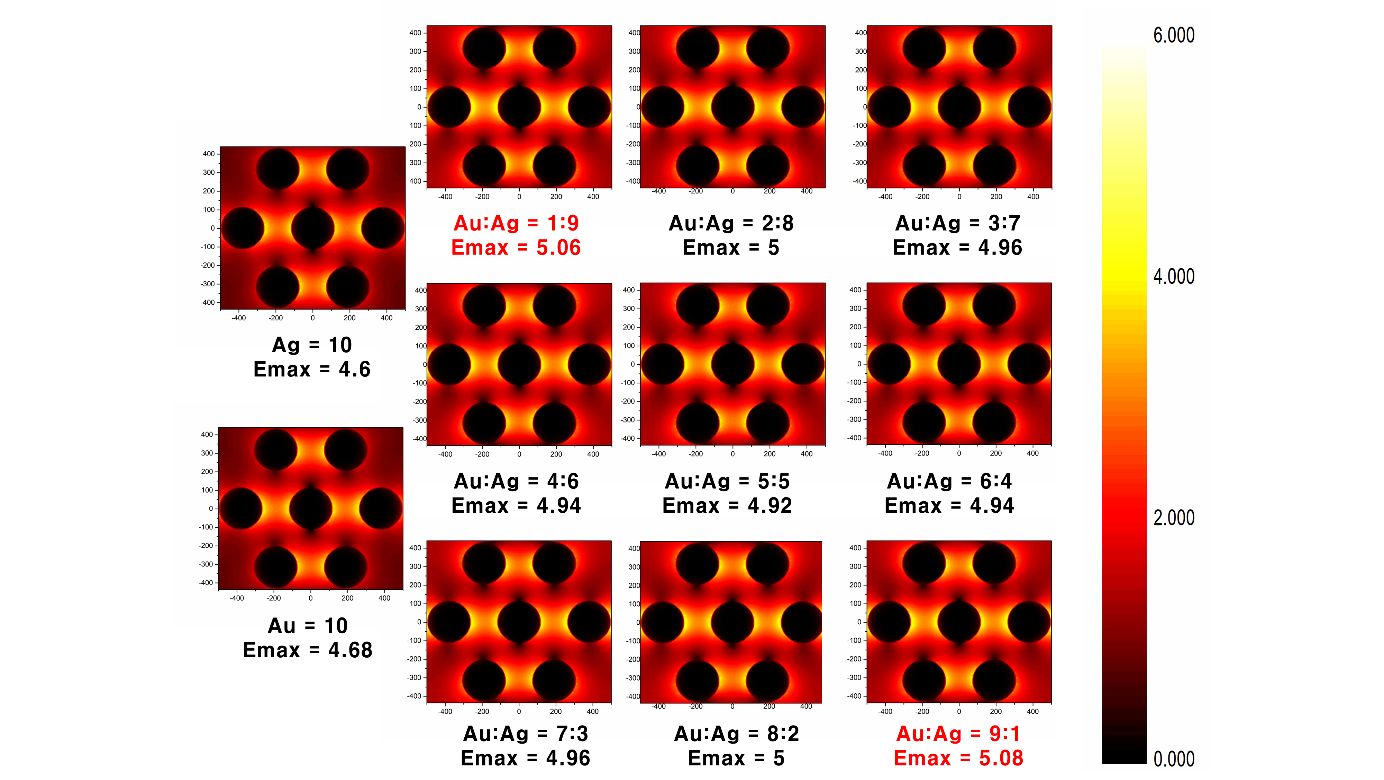


Figure S2. Numerical simulation of near-field distribution Au-Ag ratio from 10:0 to 0:10 for comparison of electric field enhancement


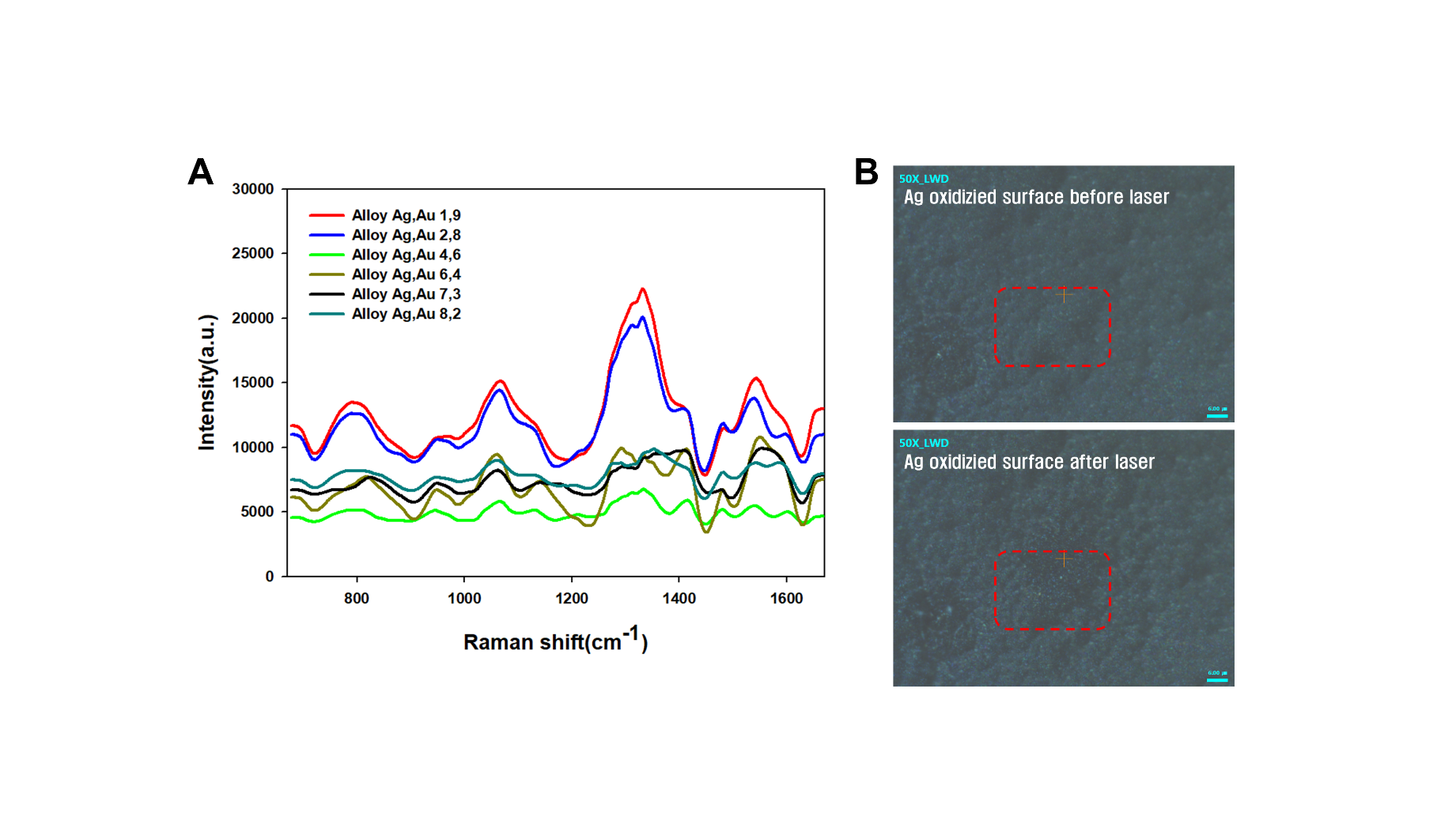


**Figure S3.** Surface oxidation of the high Ag proportion in Au-Ag alloy with Au nanogranule. A) SERS spectra of IgG-Cy3, showing the SERS signal intensity at 1321 cm⁻¹ (a characteristic peak of Cy3) for 3D AuNG@Au-AgNP substrates. B) Bright-field images of nanopillar substrates with an Au:Ag ratio of 1:9, before and after oxidation induced by laser exposure.


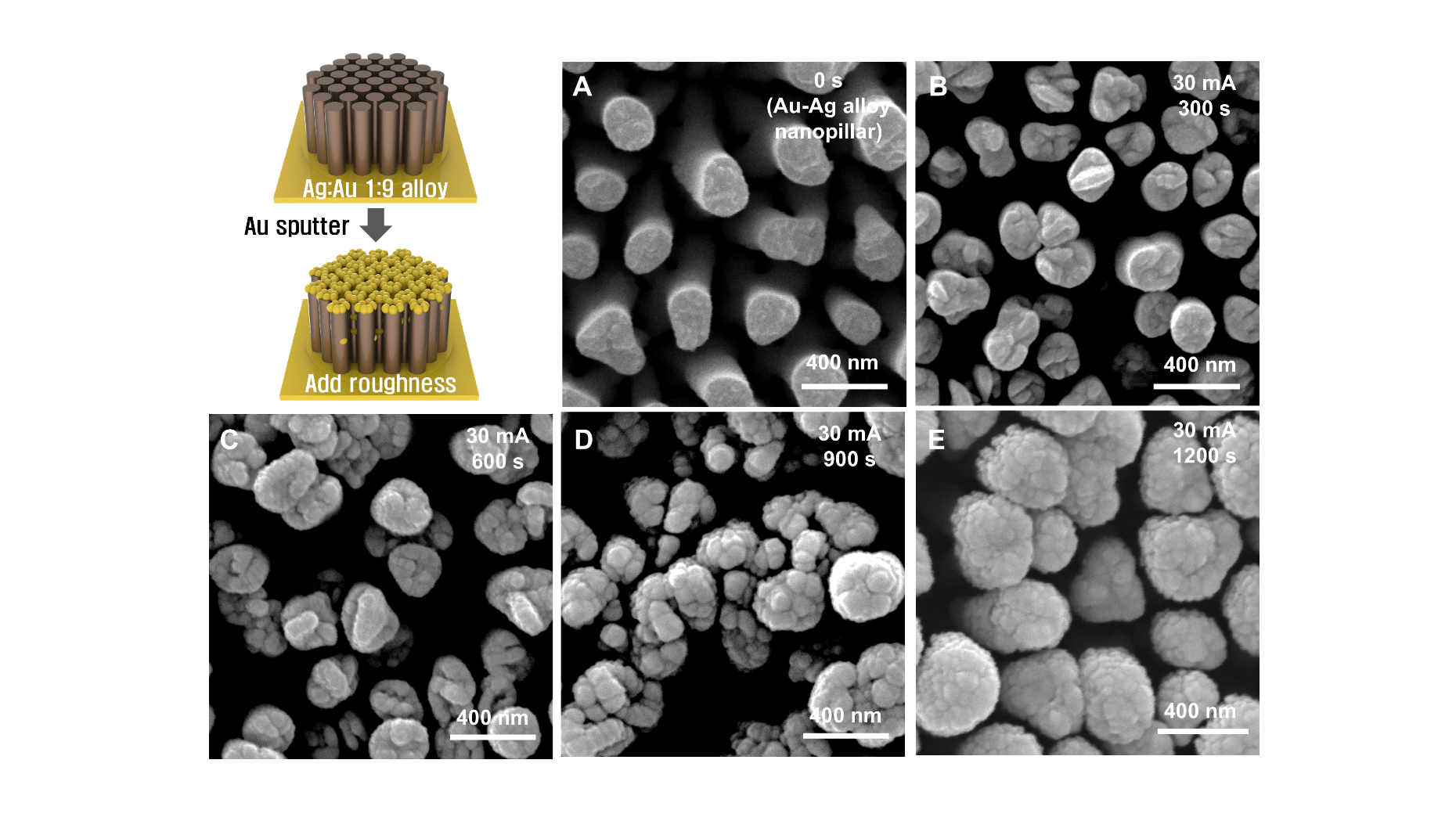


**Figure S4.** SEM images of time-dependent 3D AuNG@Au-AgNP (A) 0s, (B) 300s, (C) 600s, (D) 900s, (E) 1200s


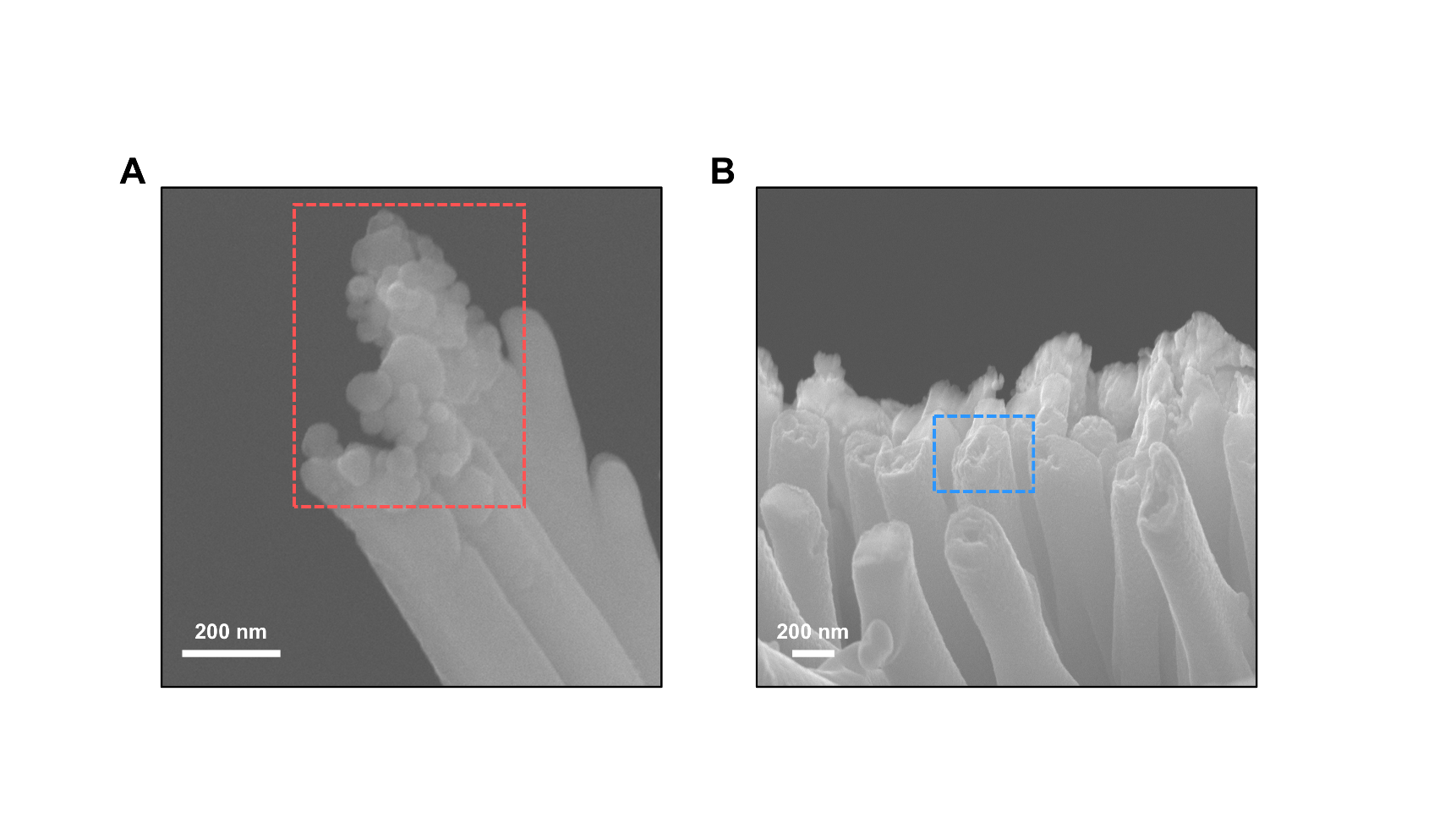


**Figure S5.** SEM images of side view of (A) AuNG@Au-AgNP and (B) Au-AgNP


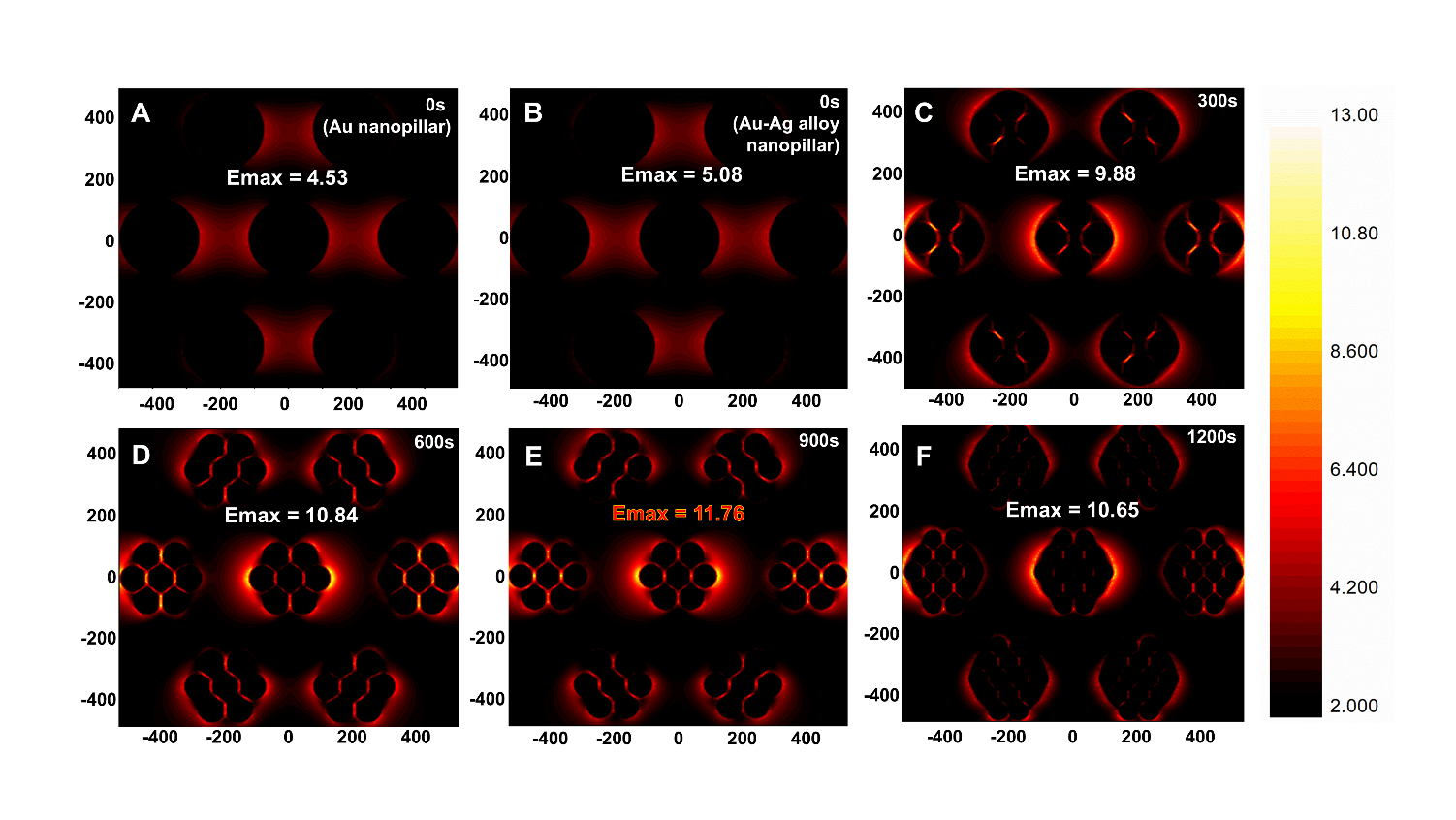


**Figure S6.** Numerical simulation of (A) AuNP, (B) Au-Ag alloy NP, time-dependent 3D AuNG@Au-AgNP (C) 300s, (D) 600s, (E) 900s, (F) 1200s


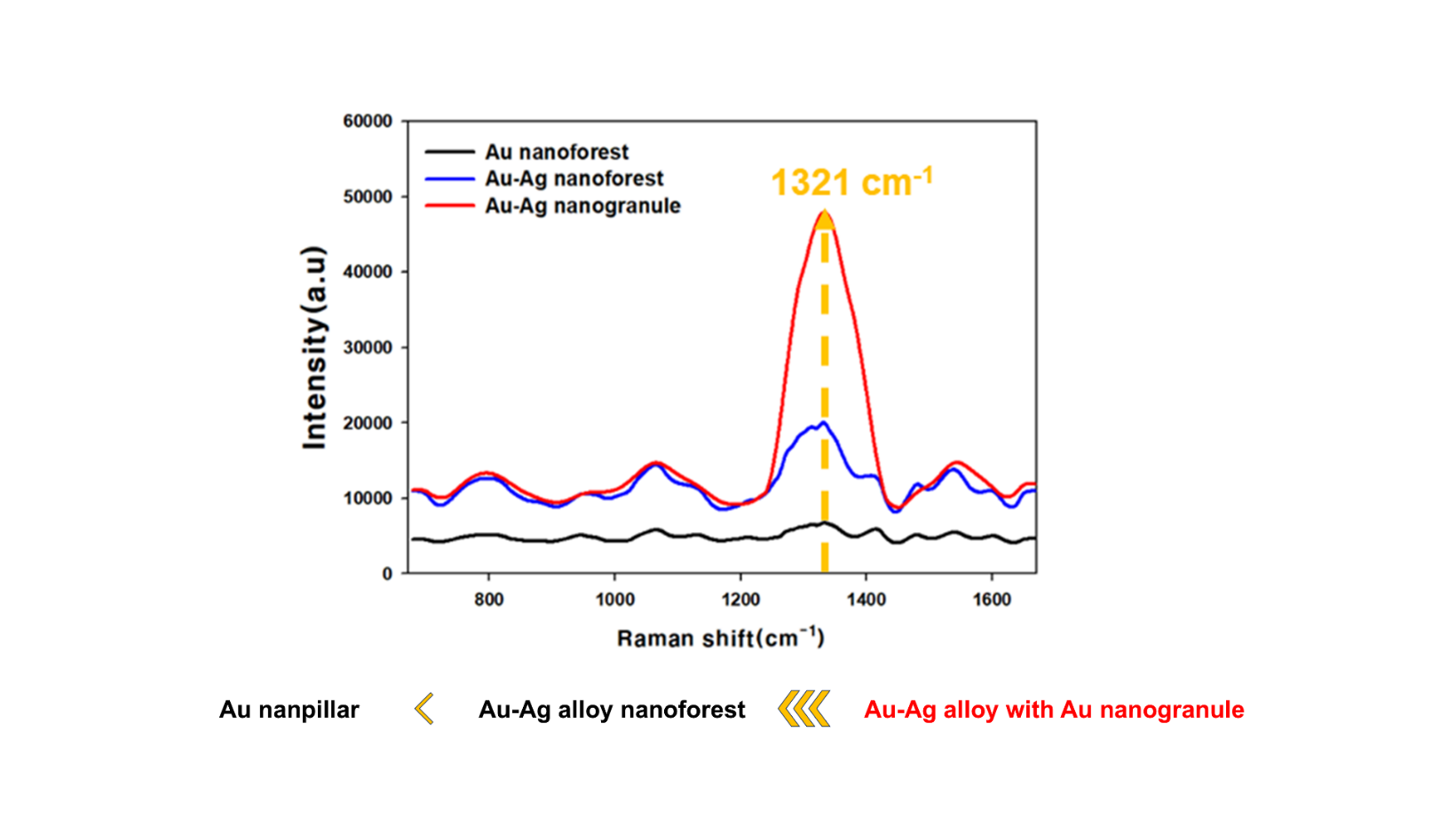


**Figure S7.** Comparison of IgG-cy3 SERS spectra for evaluating the enhancement of AuNP, Au-Ag alloy NP, 3D AuNG@Au-AgNP


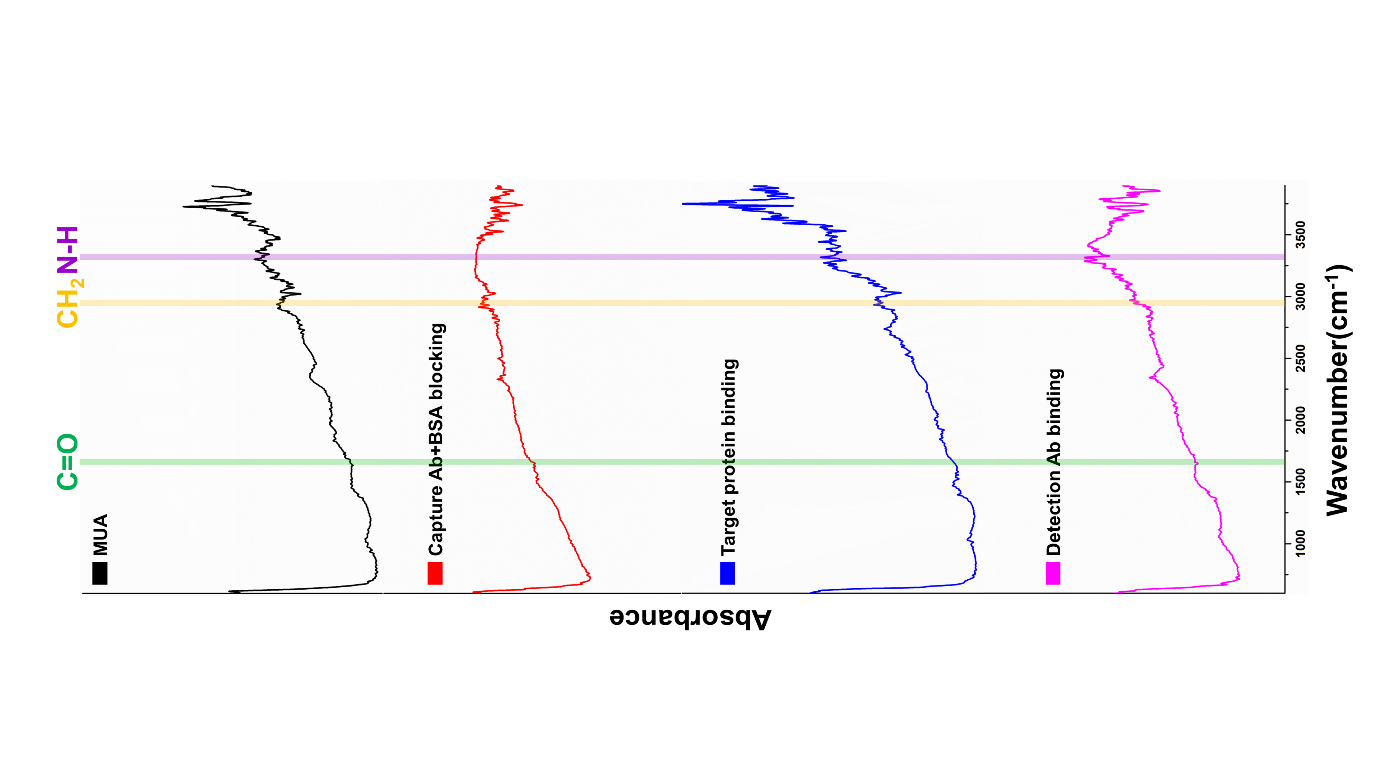


**Figure S8.** FTIR spectra of an antibody-antigen sandwich assay on the 3D AuNG@Au-AgNP based SERS biosensor


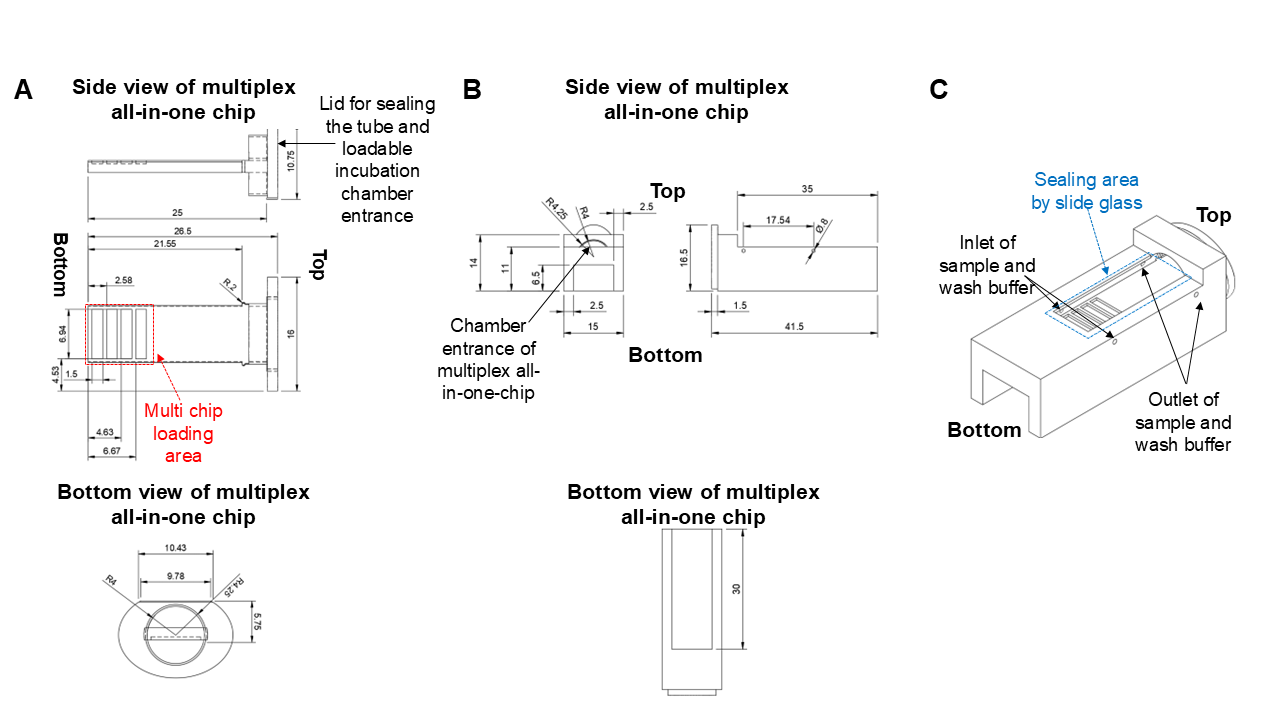


**Figure. S9.** A two-dimensional (2D) drawing of (A) multiplex all-in-one chip, (B) directly loadable incubation chamber and (C) assembly for multiplex detection (Unit: mm).


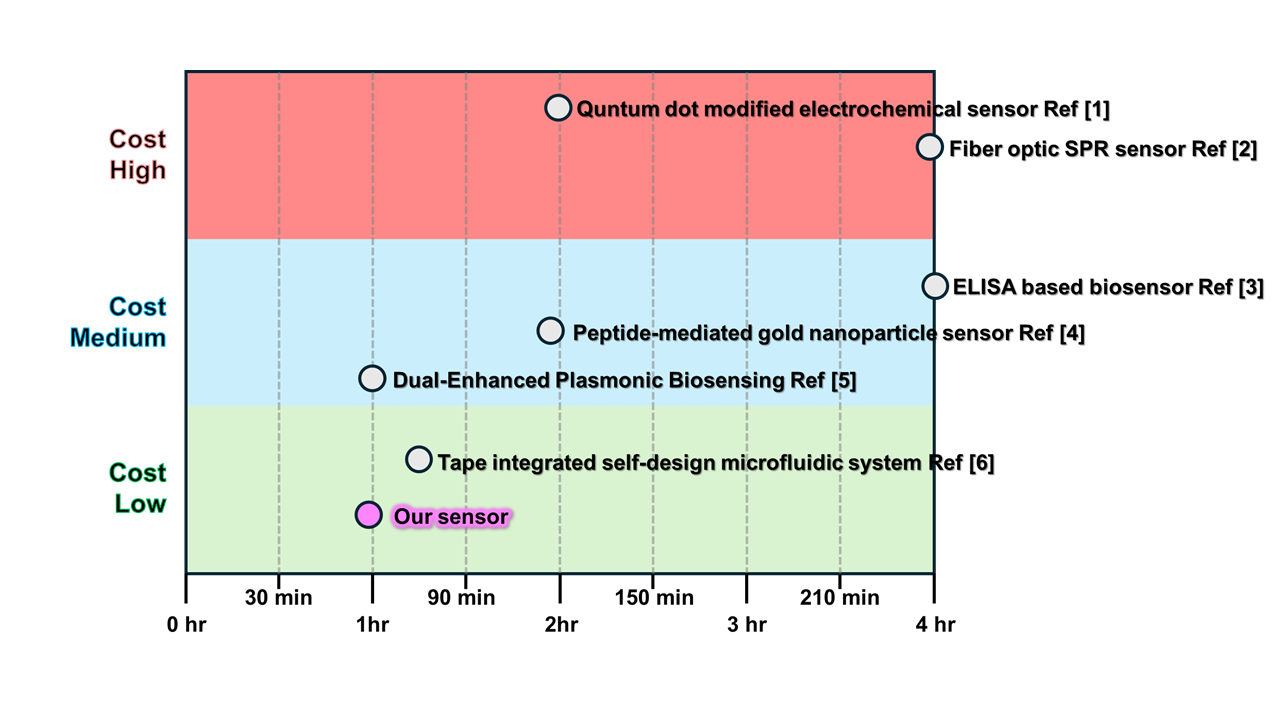


**Fig. S10**. Sensing time from incubation and cost comparison of the AuNG@Au-AgNP based SERS biosensor and other analytical tools for the detection of soluble proteins in human serum.

References

[1] A. S. Ghrera, *Anal Chim Acta* **2019**, *1056*, 26, https://doi.org/10.1016/j.aca.2018.12.047.

[2] W. Wang, Z. Mai, Y. Chen, J. Wang, L. Li, Q. Su, X. Li, X. Hong, *Sci Rep* **2017**, *7* (1), 16904, https://doi.org/10.1038/s41598-017-17276-3.

[3] X. Chen, B. Zhang, X. Song, T. Qian, X. Zheng, Y. Zhang, W. Xu, Z. Gao, L. Peng, C. Xie, *Alimentary Pharmacology & Therapeutics* **2024**, *60* (5), 593.

[4] B. Ran, W. Zheng, M. Dong, Y. Xianyu, Y. Chen, J. Wu, Z. Qian, X. Jiang, *Anal Chem* **2018**, *90* (13), 8234, https://doi.org/10.1021/acs.analchem.8b01760.

[5] L. K. Chin, J.-Y. Yang, B. Chousterman, S. Jung, D.-G. Kim, D.-H. Kim, S. Lee, C. M. Castro, R. Weissleder, S.-G. Park, *ACS nano* **2023**, *17* (4), 3610.

[6] B. Yin, C. Qian, X. Wan, A. S. M. Muhtasim Fuad Sohan, X. Lin, *Biosens Bioelectron* **2022**, *212*, 114429, https://doi.org/10.1016/j.bios.2022.114429.


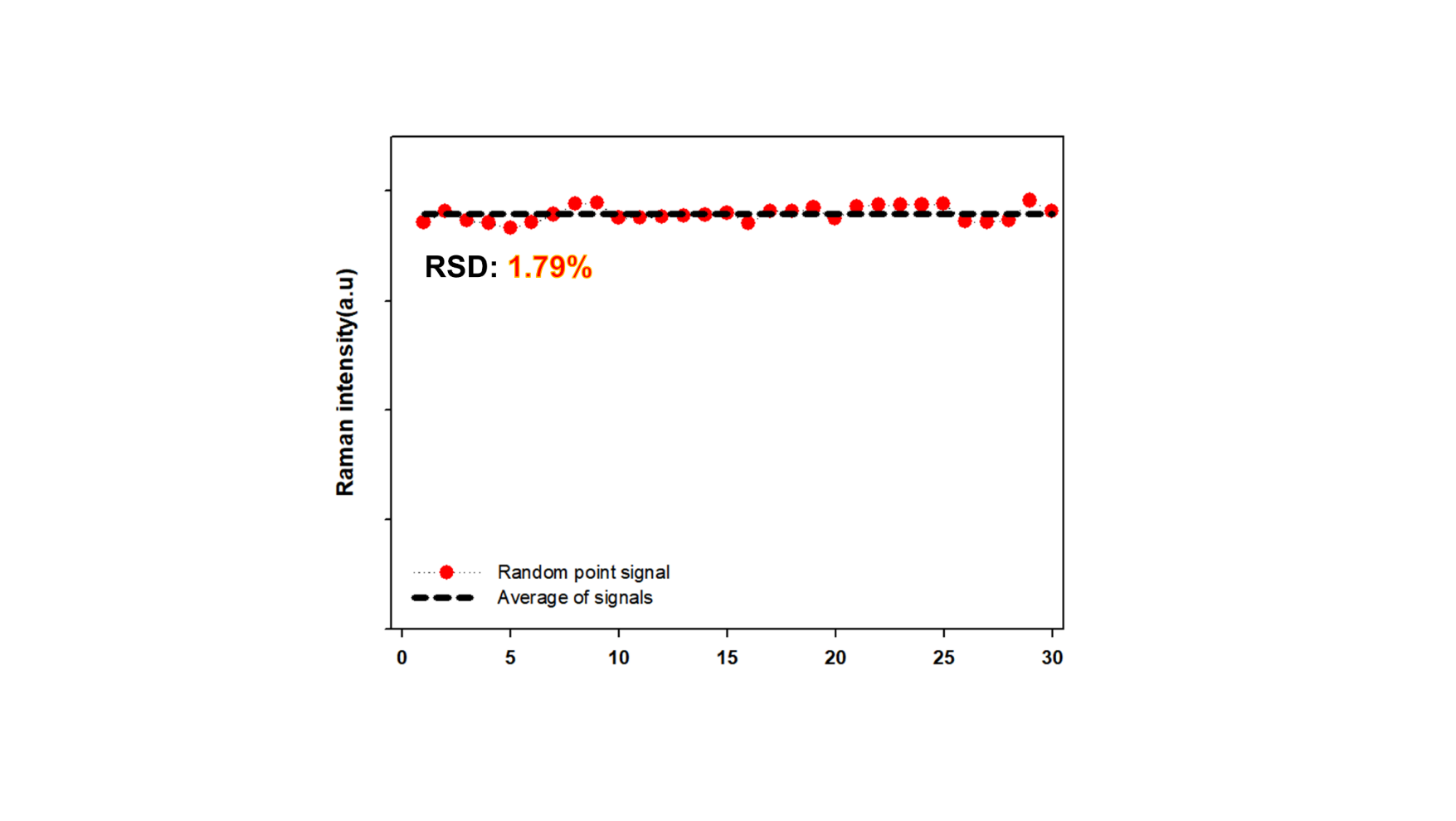


**Figure S11.** SERS signal intensities of 30 random points at 1321 cm^-1^ for detection of PD-L1 (Cy3) (RSD: 1.79%).

**
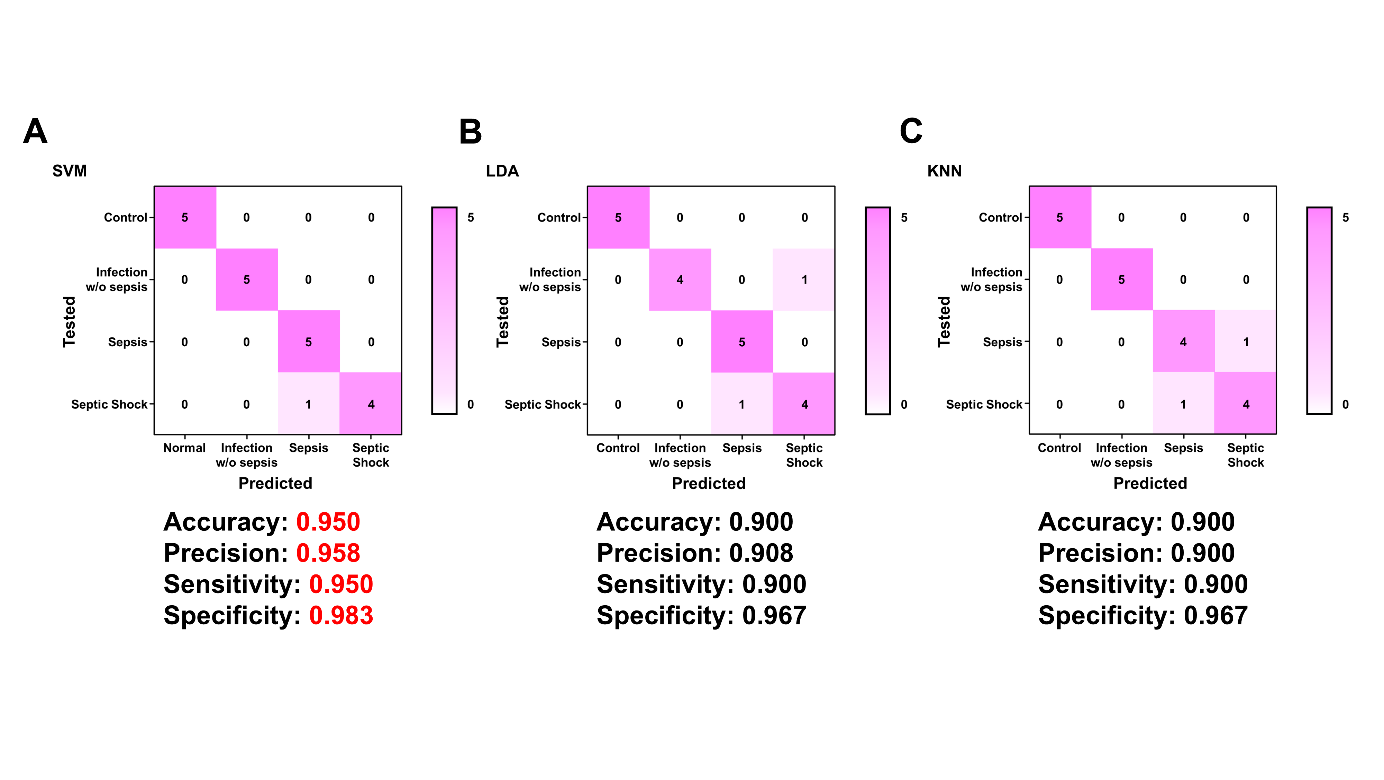
Figure S12.** Evaluation of various machine learning algorithm for applying to diagnosis

**Table S1.** Recovery test of CD123, PD-L1, ChiT, and HLA-DR in the human serum sample.

| **Samples** | **CD123** | **PD-L1** | **ChiT** | **HLA-DR** |
| --- | --- | --- | --- | --- |
| Added (pM) | 10.0 | 10.0 | 10.0 | 10.00 |
| Found (pM) | 10.7 | 10.3 | 9.69 | 11.0 |
| RSD (%) | 5.31 | 5.65 | 6.09 | 5.26 |
| Recovery (%) | 107 | 103 | 96.9 | 110 |

**Table S2.** Comparison of detection limit with other literature for soluble protein detection`.

| **Detection method** | **Target** | **Detection limit (fM)** | **Reference** |
| --- | --- | --- | --- |
| Microfluidics and Immunoassay | Proteins (Interluekin-6, procalcitonin,  C-reactive protein) | 529 fM | [1] |
| Microfluidics and Immunoassay | Proteins (Interluekin-6, procalcitonin,  C-reactive protein) | 686 fM | [2] |
| Colorimetric assay | Proteins (Interluekin-6, procalcitonin,  C-reactive protein) | 528 fM | [3] |
| Electrochemical assay | Protein (Interluekin-6) | 844 fM | [4] |
| SERS | Proteins (Interluekin-3, procalcitonin) | 100 fM | [5] |
| SERS | Proteins (Interluekin-6, procalcitonin) | 25 fM | [6] |
| **Our system**  **(SERS)** | Proteins (CD123, PD-L1, ChiT, HLA-DR) | **4-6 fM** | This work |

References

[1] B. Yin, C. Qian, X. Wan, A. S. M. Muhtasim Fuad Sohan, X. Lin, *Biosens Bioelectron* **2022**, *212*, 114429, https://doi.org/10.1016/j.bios.2022.114429.

[2] B. F. Yin, X. H. Wan, M. Z. Yang, C. C. Qian, A. Sohan, *Mil Med Res* **2022**, *9* (1), 8, https://doi.org/10.1186/s40779-022-00368-1.

[3] B. Ran, W. Zheng, M. Dong, Y. Xianyu, Y. Chen, J. Wu, Z. Qian, X. Jiang, *Anal Chem* **2018**, *90* (13), 8234, https://doi.org/10.1021/acs.analchem.8b01760.

[4] G. C. Jensen, C. E. Krause, G. A. Sotzing, J. F. Rusling, *Phys Chem Chem Phys* **2011**, *13* (11), 4888, https://doi.org/10.1039/c0cp01755h.

[5] A. Kundu, R. Rani, A. Ahmad, A. Kumar, M. Raturi, T. Gupta, R. Khan, K. S. Hazra, *Sensors & Diagnostics* **2022**, *1* (3), 449.

[6] Y. Wang, M. Guan, F. Mi, P. Geng, G. Chen, *Anal Chim Acta* **2023**, *1272*, 341523, https://doi.org/10.1016/j.aca.2023.341523.

**Table S3.** Unknown samples, healthy control, infection with and without sepsis, and septic shock patient information used for diagnosis and prognosis of sepsis.

| **Characteristics** | **Number** |
| --- | --- |
| **Healthy control** | 10 |
| Age (Median, Range) | 31 (27-38) |
| Gender (Male/Female) | 2/8 |
| **Infection w/o Sepsis** | 10 |
| Age (Median, Range) | 45 (44-94) |
| Gender (Male/Female) | 10 |
| **Sepsis** | 10 |
| Age (Median, Range) | 77.7 (53-90) |
| Gender (Male/Female) | 7/3 |
| **Septic shock** | 10 |
| Age (Median, Range) | 81.3 (68-92) |
| Gender (Male/Female) | 7/3 |
| **Unknown** | 3 |
| Age (Median, Range) | 58 (30-73) |
| Gender (Male/Female) | 2/1 |
